# (4-Butyl-1-ethyl-1,2,4-triazol-5-yl­idene)[(1,2,5,6-η)-cyclo­octa-1,5-diene](tri­phenyl­phosphane)iridium(I) tetra­fluorido­borate

**DOI:** 10.1107/S2414314624005017

**Published:** 2024-06-07

**Authors:** Timothy G. Lerch, Michael Gau, Daniel R. Albert, Edward Rajaseelan

**Affiliations:** a Department of Chemistry Millersville University,Millersville PA 17551 USA; b Department of Chemistry, University of Pennsylvania, Philadelphia, PA 19104, USA; University of Aberdeen, United Kingdom

**Keywords:** crystal structure, iridium, N-heterocyclic carbenes, cationic complexes

## Abstract

The title compound, [Ir(C_8_H_12_)(C_8_H_15_N_3_)(C_18_H_15_P)]BF_4_, a new triazole-based N-heterocyclic carbene iridium(I) cationic complex with a tetra­fluorido­borate counter-anion, crystallizes with two cations and two anions in the asymmetric unit of space group *Pc*. The Ir centers of the cations have distorted square-planar conformations, formed by a bidentate (η^2^ + η^2^) cyclo­octa-1,5-diene (COD) ligand, an N-heterocyclic carbene and a tri­phenyl­phosphane ligand with the NHC carbon atom and P atom being *cis*. In the extended structure, non-classical C–H⋯F hydrogen bonds, one of which is notably short (H⋯F = 2.21 Å), link the cations and anions. The carbon atoms of one of the COD ligands are disordered over adjacent sites in a 0.62:0.38 ratio.

## Abstract 2

The title triazole-based N-heterocyclic carbene iridium(I) cationic complex with a tetra­fluorido­borate counter-anion, crystallizes with two cations and two anions in the asymmetric unit. In the extended structure, non-classical C–H⋯F hydrogen bonds, one of which is notably short (H⋯F = 2.21 Å), link the cations and anions.

## Graphical abstract

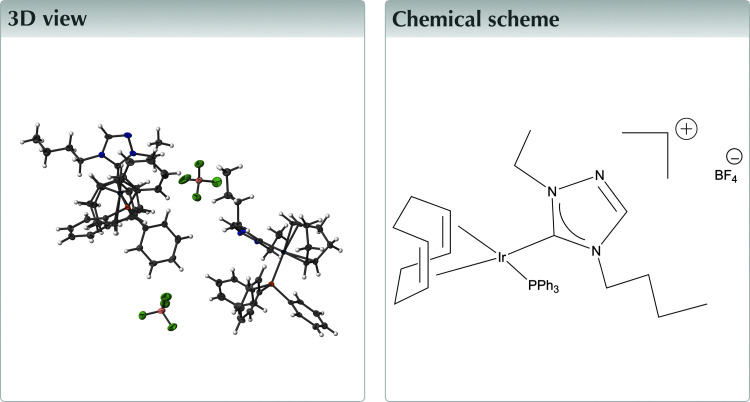

## Structure description

N-heterocyclic carbenes (NHCs) have emerged as excellent spectator ligands in homogeneous catalysis (Cazin, 2013 ▸; de Frémont *et al.*, 2009 ▸; Díez-Gonzáles *et al.*, 2009 ▸; Rovis & Nolan, 2013 ▸; Ruff *et al.*, 2016 ▸; Zuo *et al.*, 2014 ▸). Their catalytic activity in the transfer hydrogenation of ketones and imines has also been studied and reported (Albrecht *et al.*, 2002 ▸; Gnanamgari *et al.*, 2007 ▸). NHC ligands can be tuned sterically and electronically by having different substituents on the nitro­gen atoms (Gusev, 2009 ▸). Many imidazole- and triazole-based NHC rhodium and iridium complexes have been synthesized and structurally characterized (Herrmann *et al.*, 2006 ▸; Wang & Lin 1998 ▸; Chianese *et al.*, 2004 ▸). We continue to synthesize new imidazole- and triazole-based NHC complexes of rhodium and iridium, to study the effect of different substituents on the NHCs and the other ligands coordinated to the metal in transfer hydrogenation reactions (Nichol *et al.*, 2009 ▸, 2010 ▸, 2011 ▸, 2012 ▸; Idrees *et al.*, 2017*a* ▸,*b* ▸; Rood *et al.*, 2021 ▸; Rushlow *et al.*, 2021 ▸; Newman *et al.*, 2021 ▸; Castaldi *et al.*, 2021 ▸; Maynard *et al.*, 2023 ▸). The structure of the rhodium analogue of the title compound has been reported (Lerch *et al.*, 2024 ▸), and the title compound was synthesized to study the effect of the metal on catalytic properties.

**Figure 1:**
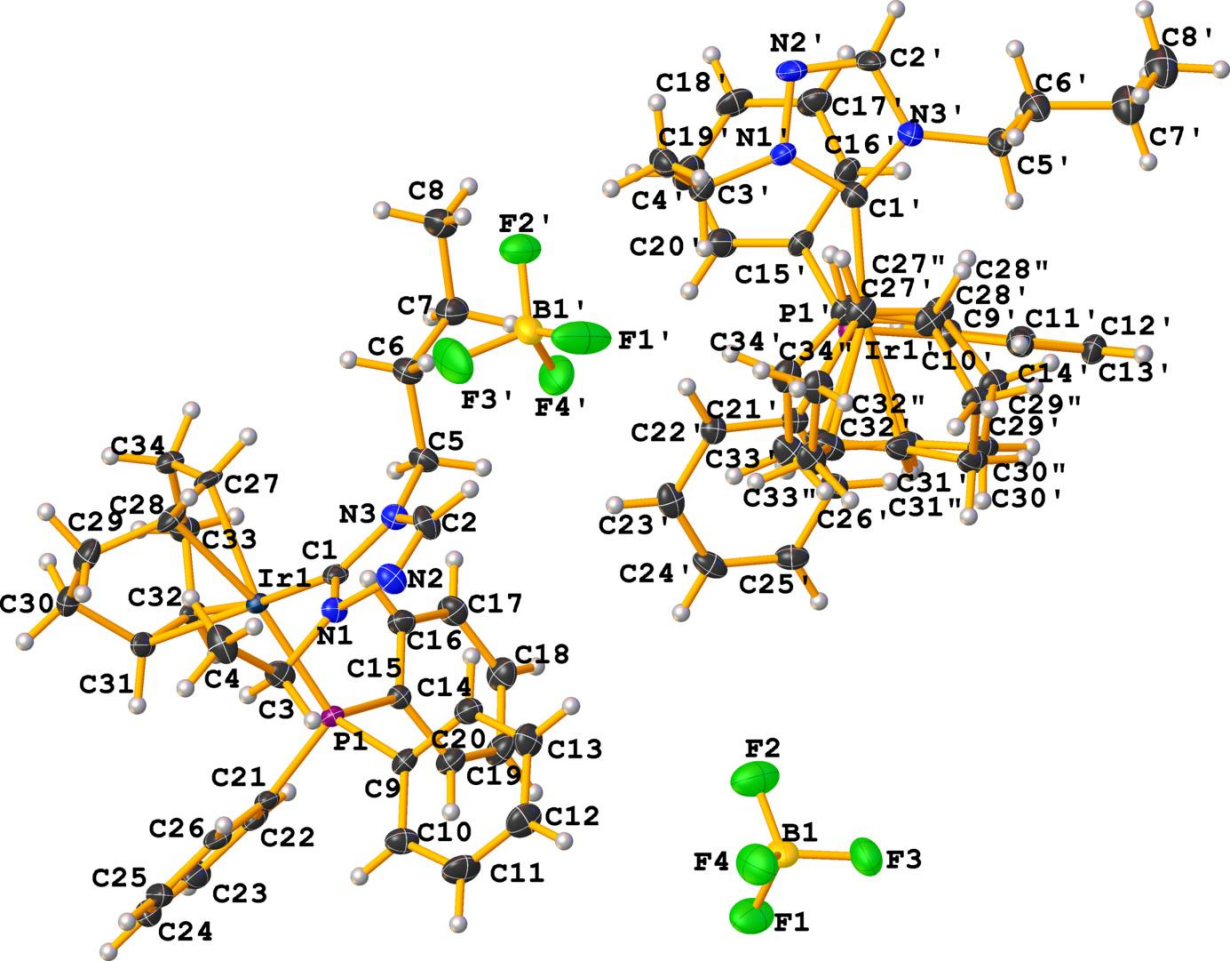
The mol­ecular entities of the title compound (**3**) with displacement ellipsoids drawn at the 50% probability level.

**Figure 2:**
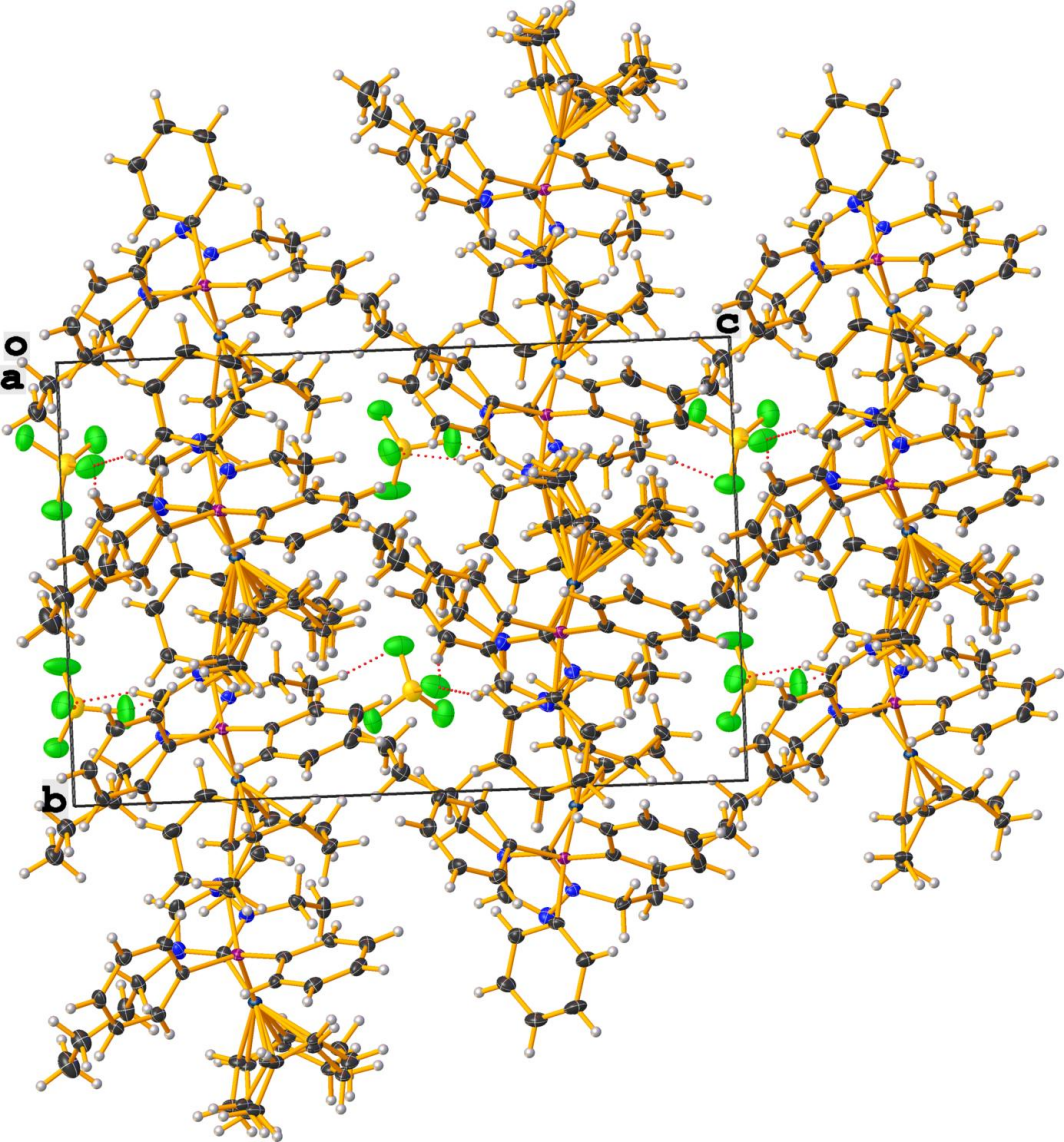
Crystal packing diagram of the title compound (**3**), viewed along [100]. C—H⋯F hydrogen bonds are shown as dotted red lines.

The mol­ecular structure of the title complex, [Ir(C_8_H_12_)(C_18_H_15_P)(C_8_H_15_N_3_)][BF_4_] (**3**), comprises an Ir^I^ cation complex and a tetra­fluorido­borate counter-anion, as shown in Fig. 1 ▸. No solvent mol­ecules are found in the structure. Two cations (*A* containing Ir1 and *B* containing Ir1′) and two anions are contained in the assymetric unit, which crystallizes in the monoclinic space group *Pc*. The distorted square-planar geometry around the iridium atoms is characterized by C1—Ir—P bond angles of 92.97 (14)° for cation *A* and 91.62 (15)° for cation *B*. The N—C—N bond angles of the NHC ligand are 102.8 (4) and 102.6 (4)° for cations *A* and *B*, respectively. The metal–phospho­rous and metal–carbene bond lengths of the title compound are similar to those of the previously published rhodium analogue (Lerch *et al.*, 2024 ▸) with Ir—C_NHC_ bond lengths of 2.035 (5) Å (cation *A*) and 2.034 (5) Å (cation *B*) and Ir—P bond lengths of 2.3145 (13) Å (cation *A*) and 2.3154 (13) Å (cation *B*). Fig. 2 ▸ shows the packing viewed along the *a* axis with non-classical C—H⋯F hydrogen bonds (Table 1 ▸) in the range of 2.21–2.49 Å for the H⋯F contacts shown as dotted red lines. The shortest C—H⋯F contacts arise from inter­actions from the CH moieties of the NHC (C2 and C2′) inter­acting with adjacent tetra­fluorido­borate anions.

## Synthesis and crystallization

**Figure 3:**
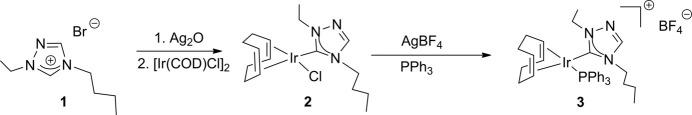
Reaction scheme for the synthesis of (**3**).

**4-Butyl-1-ethyl-1,2,4-triazolium bromide** (**1**) was synthesized by a previously published procedure (Lerch *et al.*, 2024 ▸). All other compounds used in the syntheses were obtained from Sigma-Aldrich and Strem and used as received; all syntheses were performed under a nitro­gen atmosphere. The reaction scheme is shown in Fig. 3 ▸. NMR spectra were recorded at room temperature in CDCl_3_ on a 400 MHz (operating at 100 MHz for ^13^C and 162 MHz for ^31^P) Varian spectrometer and referenced to the residual solvent peak (δ in p.p.m.). The title compound (**3**) was crystallized by slow diffusion of pentane into a CH_2_Cl­_2_ solution.

**[(1,2,5,6-η)-Cyclo­octa-1,5-diene](4-butyl-1-ethyl-1,2,4-triazol-5-yl­idene)chloro­iridium (2):** triazolium bromide (**1**) (0.070 g, 0.298 mmol) and Ag_2_O (0.035 g, 0.149 mmol) were stirred at room temperature in the dark for 1 h in CH_2_Cl_2_ (10 ml). The mixture was then filtered through Celite into [Ir(cod)Cl]_2_ (0.100 g, 0.149 mmol), and stirred again in the dark for 1.5 h. The resulting solution was filtered through Celite and the solvent was removed under reduced pressure in a rotary evaporator. The yellow solid product (**2**) was dried under vacuum. Yield: 0.145 g (99%). ^1^H NMR: δ 7.86 (*s*, 1 H, N—C3H—N), 4.75 (*q*, 2 H, N—CH_2_ of eth­yl), 4.62 (*t*, 2 H, N—CH_2_ of but­yl), 4.50 (*m*, 2 H, CH of COD), 4.48 (*m*, 2 H, CH of COD), 3.36, 3.24 (*m*, 4 H, CH_2_ of COD), 2.38, 2.08 (*m*, 4 H, CH_2_ of COD), 1.89 (*m*, 2 H, CH_2_ of but­yl), 1.55 (*m*, 2H, CH_2_ of but­yl), 1.43 (*m*, 2 H, CH_2_ of but­yl), 1.50 (*t*, 3 H, CH_3_ of eth­yl) 1.05 (*t*, 3 H, CH_3_ of but­yl). ^13^C NMR: δ 182.31 (Ir—C), 141.66 (N—C3H—N), 86.29, 85.89 (CH of COD), 48.19 (N—CH_2_ of eth­yl), 47.67 (N—CH­_2_ of but­yl), 33.81, 33.21, 32.56, 29.82 (CH_2_ of COD), 29.11 (CH­_2_ of but­yl), 19.90 (CH­_2_ of but­yl), 15.37 (CH_3_ of eth­yl), 13.61 (CH­_3_ of but­yl).

**[(1,2,5,6-η)-Cyclo­octa-1,5-diene](4-butyl-1-ethyl-1,2,4-triazol-5-yl­idene)(tri­phenyl­phosphane)iridium(I) tetra­fluorido­borate (3):** tri­phenyl­phosphane (0.0804 g, 0.307 mmol) and AgBF_4_ (0.059 g, 0.307 mmol) were added to (**2**) (0.150 g, 0.307 mmol) in CH_2_Cl_2_ (15 ml). The solution was stirred in the dark for 1.5 h. The resulting mixture was filtered through Celite and the solvent was removed under reduced pressure. The bright red-orange solid product (**3**) was dried under vacuum. Red blocks suitable for data collection were crystallized from CH_2_Cl_2_/pentane solution. Yield: 0.243 g (99%). ^1^H NMR: δ 8.19 (*s*, 1 H, N—C3H—N), 7.52-7.25 (*m*, 15 H, H_arom_), 4.41 (*q*, 2 H, N—CH_2_ of eth­yl), 4.27 (*t*, 2 H, N—CH_2_ of but­yl), 4.18 (*m*, 2 H, CH of COD), 3.96 (*m*, 2 H, CH of COD), 3.82 (*m*, 2 H, CH_2_ of COD), 3.76 (*m*, 2 H, CH_2_ of COD), 2.36 (*m*, 2 H, CH_2_ of COD), 2.26 (*m*, 2 H, CH_2_ of COD), 2.05 (*m*, CH_2_ of but­yl), 1.68 (*m*, 2 H, CH_2_ of but­yl), 1.22 (*t*, 3 H, CH_3_ of eth­yl), 0.88 (*t*, 3 H, CH_3_ of but­yl). ^13^C NMR: δ 177.39 (In—C), 143.62 (N—C3H—N), 133.63–129.04 (C_arom_), 87.24, 87.12, 86.22, 86.11 (CH of COD), 48.49 (N—CH_2_ of eth­yl), 47.66 (N—CH­_2_ of but­yl), 31.63, 31.37, 30.88, 30.85 (CH_2_ of COD), 30.42 (CH_2_ of but­yl), 19.99 (CH_2_ of but­yl), 13.85 (CH_3_ of eth­yl), 13.66 (CH_3_ of but­yl). ^31^P NMR: δ 17.56.

## Refinement

Hydrogen atoms were refined using a riding model. All carbon atoms of one of the COD ligands are disordered over adjacent sites in a 0.62:0.38 ratio. Refinement details are provided in Table 2 ▸.

## Supplementary Material

Crystal structure: contains datablock(s) I. DOI: 10.1107/S2414314624005017/hb4475sup1.cif

Structure factors: contains datablock(s) I. DOI: 10.1107/S2414314624005017/hb4475Isup2.hkl

CCDC reference: 2358743

Additional supporting information:  crystallographic information; 3D view; checkCIF report

## Figures and Tables

**Table 1:** Hydrogen-bond geometry (Å, °)

*D*—H⋯*A*	*D*—H	H⋯*A*	*D*⋯*A*	*D*—H⋯*A*
C2—H2⋯F4′	0.95	2.35	3.255 (7)	158
C4—H4*B*⋯F2^i^	0.98	2.49	3.352 (8)	147
C2′—H2′⋯F1^ii^	0.95	2.21	3.014 (6)	141
C11′—H11′⋯F4^iii^	0.95	2.46	3.363 (7)	160
C30′—H30*D*⋯F3′^iii^	0.99	2.49	3.355 (15)	146

Symmetry codes: (i) 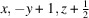; (ii) 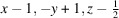; (iii) 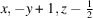.

**Table 2:** Experimental details

Crystal data	
Chemical formula	[Ir(C_8_H_12_)(C_8_H_15_N_3_)(C_18_H_15_P)]BF_4_
*M*_r_	802.68
Crystal system, space group	Monoclinic, *Pc*
Temperature (K)	100
*a*, *b*, *c* (Å)	14.1426 (3), 12.3103 (3), 18.8707 (3)
β (°)	98.492 (2)
*V* (Å^3^)	3249.36 (12)
*Z*	4
Radiation type	Mo *K*α
μ (mm^−1^)	4.21
Crystal size (mm)	0.38 × 0.21 × 0.14 × 1.90 (radius)

Data collection	
Diffractometer	Rigaku XtaLAB Synergy-S
Absorption correction	Multi-scan (CrysAlisPr; Rigaku OD, 2024 ▸)
*T*_min_, *T*_max_	0.072, 0.097
No. of measured, independent and observed [*I* > 2σ(*I*)] reflections	65731, 15859, 14407
*R*_int_	0.043
(sin θ/λ)_max_ (Å^−1^)	0.668

Refinement	
*R*[*F*^2^ > 2σ(*F*^2^)], *wR*(*F*^2^), *S*	0.027, 0.066, 1.08
No. of reflections	15859
No. of parameters	869
No. of restraints	251
H-atom treatment	H-atom parameters constrained
Δρ_max_, Δρ_min_ (e Å^−3^)	0.99, −0.79
Absolute structure	Flack *x* determined using 6483 quotients [(*I*^+^)−(*I*^−^)]/[(*I*^+^)+(*I*^−^)] (Parsons et al., 2013 ▸)
Absolute structure parameter	−0.013 (4)

Computer programs: *CrysAlis PRO* (Rigaku OD, 2024 ▸), *SHELXT* (Sheldrick, 2015*a* ▸), *SHELXL2018/3* (Sheldrick, 2015*b* ▸), *OLEX2* (Dolomanov *et al.*, 2009 ▸) and *publCIF* (Westrip, 2010 ▸).

## full crystallographic data

### (4-Butyl-1-ethyl-1,2,4-triazol-5-ylidene)[(1,2,5,6-η)-cycloocta-1,5-diene](triphenylphosphane)iridium(I) tetrafluoridoborate Crystal data

**Table:**

[Ir(C_8_H_12_)(C_8_H_15_N_3_)(C_18_H_15_P)]BF_4_	*F*(000) = 1600
*M**_r_* = 802.68	*D*_x_ = 1.641 Mg m^−^^3^
Monoclinic, *P**c*	Mo *K*α radiation, λ = 0.71073 Å
*a* = 14.1426 (3) Å	Cell parameters from 31113 reflections
*b* = 12.3103 (3) Å	θ = 2.4–28.3°
*c* = 18.8707 (3) Å	µ = 4.21 mm^−^^1^
β = 98.492 (2)°	*T* = 100 K
*V* = 3249.36 (12) Å^3^	Block, red
*Z* = 4	0.38 × 0.21 × 0.14 × 1.90 (radius) mm

### (4-Butyl-1-ethyl-1,2,4-triazol-5-ylidene)[(1,2,5,6-η)-cycloocta-1,5-diene](triphenylphosphane)iridium(I) tetrafluoridoborate Data collection

**Table:**

Rigaku XtaLAB Synergy-S diffractometer	14407 reflections with *I* > 2σ(*I*)
Detector resolution: 10.0 pixels mm^-1^	*R*_int_ = 0.043
ω scans	θ_max_ = 28.3°, θ_min_ = 2.2°
Absorption correction: multi-scan (CrysAlisPr; Rigaku OD, 2024)	*h* = −18→18
*T*_min_ = 0.072, *T*_max_ = 0.097	*k* = −16→16
65731 measured reflections	*l* = −25→25
15859 independent reflections	

### (4-Butyl-1-ethyl-1,2,4-triazol-5-ylidene)[(1,2,5,6-η)-cycloocta-1,5-diene](triphenylphosphane)iridium(I) tetrafluoridoborate Refinement

**Table:**

Refinement on *F*^2^	Hydrogen site location: inferred from neighbouring sites
Least-squares matrix: full	H-atom parameters constrained
*R*[*F*^2^ > 2σ(*F*^2^)] = 0.027	*w* = 1/[σ^2^(*F*_o_^2^) + (0.0345*P*)^2^] where *P* = (*F*_o_^2^ + 2*F*_c_^2^)/3
*wR*(*F*^2^) = 0.066	(Δ/σ)_max_ = 0.001
*S* = 1.08	Δρ_max_ = 0.99 e Å^−^^3^
15859 reflections	Δρ_min_ = −0.79 e Å^−^^3^
869 parameters	Absolute structure: Flack *x* determined using 6483 quotients [(*I*^+^)-(*I*^-^)]/[(*I*^+^)+(*I*^-^)] (Parsons et al., 2013)
251 restraints	Absolute structure parameter: −0.013 (4)

### (4-Butyl-1-ethyl-1,2,4-triazol-5-ylidene)[(1,2,5,6-η)-cycloocta-1,5-diene](triphenylphosphane)iridium(I) tetrafluoridoborate Special details

**Table:**

Geometry. All esds (except the esd in the dihedral angle between two l.s. planes) are estimated using the full covariance matrix. The cell esds are taken into account individually in the estimation of esds in distances, angles and torsion angles; correlations between esds in cell parameters are only used when they are defined by crystal symmetry. An approximate (isotropic) treatment of cell esds is used for estimating esds involving l.s. planes.

### (4-Butyl-1-ethyl-1,2,4-triazol-5-ylidene)[(1,2,5,6-η)-cycloocta-1,5-diene](triphenylphosphane)iridium(I) tetrafluoridoborate Fractional atomic coordinates and isotropic or equivalent isotropic displacement parameters (Å^2^)

**Table:**

	*x*	*y*	*z*	*U*_iso_*/*U*_eq_	Occ. (<1)
Ir1	0.56035 (2)	0.04309 (2)	0.74486 (2)	0.01394 (13)	
P1	0.68145 (9)	0.16063 (10)	0.72500 (7)	0.0144 (3)	
N1	0.4235 (3)	0.2315 (3)	0.7352 (2)	0.0178 (9)	
N2	0.3501 (3)	0.2883 (4)	0.6949 (2)	0.0237 (10)	
N3	0.3982 (3)	0.1484 (4)	0.6357 (2)	0.0195 (9)	
C1	0.4559 (3)	0.1465 (4)	0.7003 (3)	0.0181 (10)	
C2	0.3374 (4)	0.2347 (5)	0.6347 (3)	0.0251 (13)	
H2	0.291160	0.253412	0.594775	0.030*	
C3	0.4567 (4)	0.2662 (5)	0.8086 (3)	0.0226 (12)	
H3A	0.474896	0.343852	0.808720	0.027*	
H3B	0.514274	0.223878	0.828104	0.027*	
C4	0.3799 (4)	0.2502 (5)	0.8565 (3)	0.0309 (13)	
H4A	0.324126	0.295047	0.838751	0.046*	
H4B	0.405146	0.271674	0.905621	0.046*	
H4C	0.360948	0.173570	0.855754	0.046*	
C5	0.4008 (4)	0.0747 (5)	0.5747 (3)	0.0217 (11)	
H5A	0.454825	0.023354	0.586043	0.026*	
H5B	0.411380	0.117309	0.532096	0.026*	
C6	0.3068 (4)	0.0105 (5)	0.5578 (3)	0.0237 (11)	
H6A	0.302609	−0.042934	0.596377	0.028*	
H6B	0.252050	0.061010	0.556084	0.028*	
C7	0.3009 (6)	−0.0488 (5)	0.4862 (5)	0.0285 (18)	
H7A	0.308538	0.004236	0.448006	0.034*	
H7B	0.353908	−0.101911	0.488745	0.034*	
C8	0.2059 (4)	−0.1080 (5)	0.4671 (3)	0.0327 (14)	
H8A	0.205018	−0.146144	0.421448	0.049*	
H8B	0.153473	−0.055290	0.462854	0.049*	
H8C	0.198233	−0.160614	0.504826	0.049*	
C9	0.6539 (4)	0.3060 (4)	0.7094 (3)	0.0185 (11)	
C10	0.6971 (4)	0.3901 (4)	0.7508 (3)	0.0260 (12)	
H10	0.743137	0.374756	0.791564	0.031*	
C11	0.6731 (5)	0.4968 (5)	0.7327 (4)	0.0336 (14)	
H11	0.702224	0.554064	0.761837	0.040*	
C12	0.6080 (5)	0.5212 (5)	0.6736 (4)	0.0314 (14)	
H12	0.593095	0.594810	0.661705	0.038*	
C13	0.5640 (4)	0.4383 (5)	0.6311 (3)	0.0273 (13)	
H13	0.518476	0.454682	0.590241	0.033*	
C14	0.5872 (4)	0.3307 (4)	0.6489 (3)	0.0228 (11)	
H14	0.557505	0.273641	0.619841	0.027*	
C15	0.7333 (3)	0.1272 (4)	0.6451 (3)	0.0166 (10)	
C16	0.7041 (4)	0.0378 (4)	0.6029 (3)	0.0221 (12)	
H16	0.654527	−0.007690	0.614797	0.027*	
C17	0.7474 (4)	0.0143 (5)	0.5428 (3)	0.0243 (11)	
H17	0.727030	−0.047014	0.514052	0.029*	
C18	0.8203 (4)	0.0802 (6)	0.5248 (3)	0.0280 (13)	
H18	0.850013	0.063240	0.484222	0.034*	
C19	0.8495 (4)	0.1709 (5)	0.5664 (3)	0.0270 (12)	
H19	0.899201	0.215909	0.554115	0.032*	
C20	0.8063 (4)	0.1958 (4)	0.6256 (3)	0.0227 (11)	
H20	0.825371	0.258708	0.653219	0.027*	
C21	0.7818 (3)	0.1570 (4)	0.7983 (3)	0.0171 (10)	
C22	0.8659 (4)	0.0997 (4)	0.7922 (3)	0.0219 (11)	
H22	0.875969	0.070656	0.747248	0.026*	
C23	0.9346 (4)	0.0857 (5)	0.8525 (3)	0.0306 (13)	
H23	0.991603	0.046988	0.848208	0.037*	
C24	0.9212 (4)	0.1270 (5)	0.9183 (3)	0.0331 (14)	
H24	0.968844	0.117353	0.958920	0.040*	
C25	0.8375 (4)	0.1830 (5)	0.9248 (3)	0.0312 (14)	
H25	0.828036	0.211766	0.969933	0.037*	
C26	0.7677 (4)	0.1969 (5)	0.8656 (3)	0.0226 (11)	
H26	0.709971	0.233601	0.870706	0.027*	
C27	0.4678 (5)	−0.1004 (5)	0.7291 (4)	0.0213 (14)	
H27	0.415887	−0.093570	0.687203	0.026*	
C28	0.4476 (5)	−0.0460 (4)	0.7894 (4)	0.0249 (13)	
H28	0.384238	−0.008361	0.782477	0.030*	
C29	0.4832 (4)	−0.0762 (5)	0.8664 (3)	0.0286 (13)	
H29E	0.442895	−0.135357	0.881104	0.034*	
H29F	0.476632	−0.012652	0.897421	0.034*	
C30	0.5881 (4)	−0.1133 (5)	0.8775 (3)	0.0248 (12)	
H30E	0.615473	−0.103752	0.928491	0.030*	
H30F	0.590902	−0.191575	0.865930	0.030*	
C31	0.6483 (4)	−0.0496 (4)	0.8308 (3)	0.0216 (12)	
H31	0.700609	−0.005889	0.858790	0.026*	
C32	0.6697 (4)	−0.0858 (5)	0.7652 (4)	0.0178 (13)	
H32	0.734469	−0.064091	0.754908	0.021*	
C33	0.6311 (4)	−0.1877 (4)	0.7278 (3)	0.0220 (11)	
H33E	0.666546	−0.250783	0.750978	0.026*	
H33F	0.642737	−0.185116	0.677416	0.026*	
C34	0.5240 (4)	−0.2050 (4)	0.7289 (3)	0.0196 (11)	
H34E	0.497327	−0.248288	0.686405	0.024*	
H34F	0.515634	−0.247666	0.772053	0.024*	
Ir1'	0.06571 (2)	0.45638 (2)	0.25589 (2)	0.01456 (14)	
P1'	0.19170 (9)	0.34777 (10)	0.23359 (7)	0.0147 (3)	
N1'	−0.0612 (3)	0.2590 (4)	0.2479 (2)	0.0202 (9)	
N2'	−0.1265 (3)	0.1937 (4)	0.2068 (3)	0.0275 (11)	
N3'	−0.0829 (3)	0.3325 (4)	0.1449 (2)	0.0235 (10)	
C1'	−0.0316 (4)	0.3448 (4)	0.2119 (3)	0.0191 (11)	
C2'	−0.1377 (4)	0.2405 (5)	0.1444 (3)	0.0267 (13)	
H2'	−0.178674	0.214263	0.103547	0.032*	
C3'	−0.0364 (4)	0.2351 (5)	0.3238 (3)	0.0229 (11)	
H3'A	0.015316	0.284542	0.345173	0.027*	
H3'B	−0.012376	0.159638	0.329881	0.027*	
C4'	−0.1231 (4)	0.2488 (5)	0.3627 (3)	0.0274 (12)	
H4'A	−0.148981	0.322296	0.354497	0.041*	
H4'B	−0.103700	0.237281	0.414186	0.041*	
H4'C	−0.172161	0.195523	0.344362	0.041*	
C5'	−0.0823 (4)	0.4058 (5)	0.0832 (3)	0.0253 (12)	
H5'A	−0.066755	0.363875	0.041670	0.030*	
H5'B	−0.032243	0.461869	0.095192	0.030*	
C6'	−0.1796 (4)	0.4609 (5)	0.0633 (3)	0.0296 (14)	
H6'A	−0.191721	0.508856	0.103164	0.036*	
H6'B	−0.230223	0.404738	0.056874	0.036*	
C7'	−0.1851 (5)	0.5282 (6)	−0.0054 (4)	0.0348 (16)	
H7'A	−0.135748	0.585818	0.001292	0.042*	
H7'B	−0.171774	0.480858	−0.045187	0.042*	
C8'	−0.2834 (5)	0.5799 (6)	−0.0249 (4)	0.0426 (17)	
H8'A	−0.331822	0.522864	−0.034686	0.064*	
H8'B	−0.283923	0.625312	−0.067658	0.064*	
H8'C	−0.297515	0.624956	0.015042	0.064*	
C9'	0.2347 (3)	0.3836 (4)	0.1503 (3)	0.0166 (10)	
C10'	0.3040 (4)	0.3168 (4)	0.1259 (3)	0.0210 (11)	
H10'	0.325563	0.253092	0.151765	0.025*	
C11'	0.3407 (4)	0.3439 (5)	0.0641 (3)	0.0235 (11)	
H11'	0.388281	0.299432	0.048161	0.028*	
C12'	0.3078 (5)	0.4364 (6)	0.0250 (4)	0.0254 (15)	
H12'	0.332872	0.454839	−0.017480	0.030*	
C13'	0.2384 (4)	0.5013 (4)	0.0486 (3)	0.0229 (11)	
H13'	0.215555	0.563705	0.021754	0.028*	
C14'	0.2018 (4)	0.4757 (5)	0.1112 (3)	0.0221 (11)	
H14'	0.154755	0.520842	0.127183	0.026*	
C15'	0.1671 (4)	0.2016 (4)	0.2207 (3)	0.0175 (11)	
C16'	0.1112 (4)	0.1715 (4)	0.1558 (3)	0.0232 (11)	
H16'	0.091620	0.225043	0.120395	0.028*	
C17'	0.0846 (5)	0.0642 (5)	0.1430 (4)	0.0301 (14)	
H17'	0.047137	0.044280	0.098959	0.036*	
C18'	0.1126 (5)	−0.0143 (5)	0.1948 (4)	0.0308 (14)	
H18'	0.093809	−0.087787	0.186235	0.037*	
C19'	0.1675 (5)	0.0144 (5)	0.2583 (4)	0.0357 (15)	
H19'	0.186661	−0.039504	0.293520	0.043*	
C20'	0.1952 (4)	0.1220 (5)	0.2715 (3)	0.0277 (12)	
H20'	0.233472	0.140985	0.315452	0.033*	
C21'	0.2975 (3)	0.3578 (4)	0.3019 (3)	0.0173 (10)	
C22'	0.2909 (4)	0.3235 (4)	0.3713 (3)	0.0200 (11)	
H22'	0.233396	0.291154	0.381357	0.024*	
C23'	0.3676 (4)	0.3361 (5)	0.4263 (3)	0.0252 (12)	
H23'	0.362911	0.310130	0.473050	0.030*	
C24'	0.4511 (4)	0.3868 (5)	0.4126 (3)	0.0253 (12)	
H24'	0.503594	0.395295	0.449838	0.030*	
C25'	0.4570 (4)	0.4248 (5)	0.3442 (3)	0.0241 (12)	
H25'	0.513378	0.460594	0.334876	0.029*	
C26'	0.3808 (4)	0.4109 (4)	0.2891 (3)	0.0196 (10)	
H26'	0.385426	0.437649	0.242504	0.023*	
C27"	−0.054 (2)	0.5546 (19)	0.2923 (15)	0.0225 (17)	0.38
H27"	−0.116351	0.514980	0.290877	0.027*	0.38
C27'	−0.0481 (13)	0.5362 (10)	0.3006 (8)	0.0217 (16)	0.62
H27'	−0.109433	0.494533	0.294132	0.026*	0.62
C28"	−0.034 (3)	0.588 (3)	0.2269 (16)	0.0219 (18)	0.38
H28"	−0.084793	0.569968	0.186116	0.026*	0.38
C28'	−0.0329 (16)	0.5953 (14)	0.2398 (9)	0.0216 (17)	0.62
H28'	−0.085030	0.586381	0.198175	0.026*	0.62
C29"	0.0205 (13)	0.6881 (15)	0.2153 (9)	0.0220 (18)	0.38
H29A	0.004551	0.708284	0.164119	0.026*	0.38
H29B	−0.003498	0.747049	0.243535	0.026*	0.38
C29'	0.0196 (8)	0.7046 (8)	0.2404 (6)	0.0212 (15)	0.62
H29C	−0.002983	0.742430	0.194761	0.025*	0.62
H29D	0.000696	0.749514	0.279532	0.025*	0.62
C30"	0.1251 (17)	0.686 (2)	0.2331 (11)	0.0230 (18)	0.38
H30A	0.145113	0.750808	0.262343	0.028*	0.38
H30B	0.152284	0.692691	0.187864	0.028*	0.38
C30'	0.1255 (10)	0.6997 (12)	0.2498 (7)	0.0252 (17)	0.62
H30C	0.151652	0.760385	0.281158	0.030*	0.62
H30D	0.146704	0.710325	0.202545	0.030*	0.62
C31"	0.169 (4)	0.589 (3)	0.2720 (18)	0.0231 (18)	0.38
H31"	0.235211	0.570753	0.262864	0.028*	0.38
C31'	0.167 (2)	0.5931 (18)	0.2821 (10)	0.0227 (17)	0.62
H31'	0.232495	0.577334	0.270117	0.027*	0.62
C32"	0.148 (3)	0.560 (2)	0.341 (2)	0.0235 (18)	0.38
H32"	0.200620	0.520543	0.372173	0.028*	0.38
C32'	0.1504 (17)	0.5444 (13)	0.3434 (12)	0.0228 (16)	0.62
H32'	0.206080	0.502396	0.368431	0.027*	0.62
C33"	0.0839 (15)	0.6266 (14)	0.3833 (12)	0.0244 (17)	0.38
H33A	0.100972	0.609298	0.434805	0.029*	0.38
H33B	0.098176	0.704393	0.377044	0.029*	0.38
C33'	0.0888 (8)	0.5962 (9)	0.3936 (7)	0.0285 (16)	0.62
H33C	0.113197	0.573772	0.443275	0.034*	0.62
H33D	0.095183	0.676165	0.390951	0.034*	0.62
C34"	−0.0181 (12)	0.6105 (13)	0.3634 (9)	0.0252 (17)	0.38
H34A	−0.049088	0.682631	0.362734	0.030*	0.38
H34B	−0.040938	0.567779	0.401957	0.030*	0.38
C34'	−0.0131 (7)	0.5677 (9)	0.3776 (6)	0.0246 (15)	0.62
H34C	−0.051177	0.630404	0.390122	0.030*	0.62
H34D	−0.025590	0.506407	0.408899	0.030*	0.62
F1	0.6760 (3)	0.7630 (3)	0.5436 (3)	0.0520 (11)	
F2	0.5460 (3)	0.6681 (3)	0.4912 (3)	0.0522 (11)	
F3	0.5814 (3)	0.8334 (3)	0.4480 (2)	0.0428 (9)	
F4	0.5261 (3)	0.8233 (3)	0.5531 (2)	0.0504 (10)	
B1	0.5832 (5)	0.7701 (6)	0.5084 (4)	0.0310 (15)	
F1'	0.0448 (3)	0.3162 (3)	0.4888 (3)	0.0691 (16)	
F2'	0.0456 (3)	0.1342 (3)	0.4753 (2)	0.0416 (9)	
F3'	0.1072 (3)	0.2088 (4)	0.5808 (2)	0.0597 (12)	
F4'	0.1850 (3)	0.2295 (3)	0.4875 (2)	0.0460 (10)	
B1'	0.0940 (5)	0.2228 (6)	0.5074 (4)	0.0288 (14)	

### (4-Butyl-1-ethyl-1,2,4-triazol-5-ylidene)[(1,2,5,6-η)-cycloocta-1,5-diene](triphenylphosphane)iridium(I) tetrafluoridoborate Atomic displacement parameters (Å^2^)

**Table:**

	*U*^11^	*U*^22^	*U*^33^	*U*^12^	*U*^13^	*U*^23^
Ir1	0.01192 (18)	0.01183 (12)	0.0180 (4)	−0.00071 (9)	0.0020 (2)	0.00016 (10)
P1	0.0139 (6)	0.0127 (6)	0.0169 (7)	−0.0010 (5)	0.0028 (5)	−0.0006 (5)
N1	0.015 (2)	0.016 (2)	0.021 (2)	0.0039 (16)	0.0011 (17)	0.0001 (18)
N2	0.020 (2)	0.026 (2)	0.024 (2)	0.0069 (18)	0.0009 (18)	−0.0012 (19)
N3	0.019 (2)	0.020 (2)	0.020 (2)	0.0008 (17)	0.0025 (17)	−0.0019 (17)
C1	0.014 (2)	0.017 (3)	0.022 (3)	−0.0033 (19)	−0.0005 (19)	0.001 (2)
C2	0.019 (3)	0.032 (3)	0.023 (3)	0.011 (2)	−0.001 (2)	0.005 (3)
C3	0.025 (3)	0.018 (3)	0.024 (3)	0.004 (2)	0.001 (2)	−0.005 (2)
C4	0.028 (3)	0.041 (4)	0.024 (3)	0.013 (3)	0.006 (2)	0.002 (3)
C5	0.019 (3)	0.027 (3)	0.019 (3)	−0.001 (2)	0.002 (2)	−0.005 (2)
C6	0.020 (3)	0.028 (3)	0.023 (3)	0.000 (2)	0.001 (2)	−0.003 (2)
C7	0.025 (4)	0.030 (4)	0.030 (4)	−0.003 (2)	0.003 (3)	−0.002 (3)
C8	0.034 (3)	0.035 (3)	0.028 (3)	−0.006 (3)	0.000 (2)	−0.004 (3)
C9	0.019 (3)	0.014 (3)	0.024 (3)	−0.001 (2)	0.008 (2)	0.001 (2)
C10	0.023 (3)	0.020 (3)	0.033 (3)	0.000 (2)	0.001 (2)	−0.005 (2)
C11	0.038 (3)	0.017 (3)	0.045 (4)	−0.003 (2)	0.003 (3)	−0.007 (3)
C12	0.033 (3)	0.018 (3)	0.044 (4)	0.005 (2)	0.008 (3)	0.000 (3)
C13	0.027 (3)	0.023 (3)	0.031 (3)	0.007 (2)	0.003 (2)	0.005 (2)
C14	0.024 (3)	0.021 (3)	0.024 (3)	−0.003 (2)	0.004 (2)	−0.001 (2)
C15	0.016 (2)	0.016 (2)	0.017 (2)	0.0013 (19)	0.0024 (18)	0.000 (2)
C16	0.023 (3)	0.019 (3)	0.023 (3)	−0.002 (2)	0.001 (2)	−0.001 (2)
C17	0.028 (3)	0.025 (3)	0.021 (3)	0.002 (2)	0.005 (2)	−0.005 (2)
C18	0.031 (3)	0.031 (3)	0.025 (3)	0.000 (3)	0.013 (2)	−0.001 (3)
C19	0.030 (3)	0.024 (3)	0.030 (3)	−0.004 (2)	0.016 (2)	0.000 (2)
C20	0.023 (3)	0.020 (3)	0.026 (3)	−0.002 (2)	0.008 (2)	−0.002 (2)
C21	0.016 (2)	0.014 (2)	0.021 (2)	−0.0045 (18)	0.0010 (19)	0.0007 (19)
C22	0.018 (2)	0.017 (3)	0.030 (3)	−0.005 (2)	0.003 (2)	0.006 (2)
C23	0.022 (3)	0.028 (3)	0.038 (3)	−0.003 (2)	−0.008 (2)	0.011 (3)
C24	0.032 (3)	0.035 (3)	0.028 (3)	−0.013 (3)	−0.007 (2)	0.011 (3)
C25	0.037 (3)	0.035 (3)	0.020 (3)	−0.019 (3)	−0.002 (2)	0.002 (2)
C26	0.023 (3)	0.022 (3)	0.024 (3)	−0.011 (2)	0.005 (2)	−0.001 (2)
C27	0.018 (3)	0.015 (3)	0.031 (4)	−0.009 (2)	0.001 (3)	0.002 (3)
C28	0.020 (3)	0.020 (3)	0.037 (3)	−0.006 (2)	0.012 (2)	0.005 (2)
C29	0.033 (3)	0.025 (3)	0.030 (3)	−0.004 (2)	0.013 (3)	0.006 (2)
C30	0.029 (3)	0.021 (3)	0.024 (3)	−0.001 (2)	0.003 (2)	0.008 (2)
C31	0.022 (3)	0.015 (3)	0.025 (3)	0.0009 (19)	−0.005 (2)	0.005 (2)
C32	0.015 (3)	0.011 (3)	0.026 (4)	0.002 (2)	−0.002 (2)	0.002 (2)
C33	0.021 (3)	0.012 (2)	0.031 (3)	0.0022 (19)	−0.002 (2)	0.000 (2)
C34	0.017 (2)	0.016 (3)	0.026 (3)	−0.0027 (19)	0.002 (2)	−0.001 (2)
Ir1'	0.0130 (2)	0.01289 (12)	0.0178 (4)	0.00096 (9)	0.0025 (2)	0.00014 (10)
P1'	0.0139 (6)	0.0129 (6)	0.0176 (7)	0.0016 (5)	0.0036 (5)	0.0015 (5)
N1'	0.021 (2)	0.019 (2)	0.021 (2)	−0.0064 (17)	0.0040 (18)	−0.0006 (18)
N2'	0.026 (2)	0.031 (3)	0.025 (2)	−0.015 (2)	0.0024 (19)	−0.004 (2)
N3'	0.022 (2)	0.027 (2)	0.020 (2)	−0.0055 (19)	0.0001 (18)	0.0025 (19)
C1'	0.016 (2)	0.022 (3)	0.020 (2)	0.001 (2)	0.004 (2)	−0.001 (2)
C2'	0.021 (3)	0.036 (4)	0.023 (3)	−0.015 (2)	0.002 (2)	−0.004 (3)
C3'	0.021 (3)	0.023 (3)	0.025 (3)	−0.003 (2)	0.002 (2)	0.005 (2)
C4'	0.024 (3)	0.039 (3)	0.019 (3)	−0.005 (2)	0.003 (2)	−0.001 (2)
C5'	0.022 (3)	0.034 (3)	0.020 (3)	−0.005 (2)	0.002 (2)	0.006 (2)
C6'	0.019 (3)	0.046 (4)	0.024 (3)	−0.001 (2)	0.004 (2)	0.003 (3)
C7'	0.025 (3)	0.045 (4)	0.035 (4)	0.008 (3)	0.007 (3)	0.008 (3)
C8'	0.029 (3)	0.060 (5)	0.040 (4)	0.008 (3)	0.006 (3)	0.015 (3)
C9'	0.016 (2)	0.018 (2)	0.016 (2)	0.0016 (19)	0.0038 (18)	0.0006 (19)
C10'	0.020 (3)	0.019 (3)	0.024 (3)	0.001 (2)	0.004 (2)	0.002 (2)
C11'	0.024 (3)	0.024 (3)	0.024 (3)	0.000 (2)	0.008 (2)	−0.003 (2)
C12'	0.029 (4)	0.030 (3)	0.018 (3)	−0.007 (3)	0.008 (3)	0.002 (3)
C13'	0.028 (3)	0.019 (3)	0.021 (3)	−0.002 (2)	0.004 (2)	0.004 (2)
C14'	0.023 (3)	0.021 (3)	0.022 (3)	0.001 (2)	0.000 (2)	0.000 (2)
C15'	0.018 (2)	0.012 (3)	0.024 (3)	0.0008 (19)	0.008 (2)	0.001 (2)
C16'	0.029 (3)	0.017 (2)	0.024 (3)	−0.003 (2)	0.006 (2)	0.001 (2)
C17'	0.036 (3)	0.022 (3)	0.033 (3)	−0.006 (2)	0.007 (3)	−0.007 (2)
C18'	0.035 (3)	0.013 (3)	0.045 (4)	−0.004 (2)	0.007 (3)	−0.004 (3)
C19'	0.042 (4)	0.017 (3)	0.046 (4)	0.001 (3)	0.002 (3)	0.009 (3)
C20'	0.027 (3)	0.023 (3)	0.031 (3)	0.001 (2)	−0.002 (2)	0.001 (2)
C21'	0.016 (2)	0.013 (2)	0.023 (2)	0.0050 (18)	0.0019 (19)	−0.0011 (19)
C22'	0.019 (3)	0.019 (3)	0.022 (3)	0.005 (2)	0.004 (2)	0.002 (2)
C23'	0.029 (3)	0.023 (3)	0.023 (3)	0.012 (2)	0.004 (2)	0.002 (2)
C24'	0.021 (3)	0.027 (3)	0.025 (3)	0.007 (2)	−0.005 (2)	−0.008 (2)
C25'	0.017 (3)	0.024 (3)	0.031 (3)	−0.001 (2)	0.004 (2)	−0.007 (2)
C26'	0.020 (3)	0.018 (2)	0.020 (2)	0.002 (2)	0.002 (2)	−0.004 (2)
C27"	0.021 (3)	0.017 (4)	0.032 (4)	0.007 (3)	0.009 (3)	0.000 (3)
C27'	0.020 (3)	0.016 (3)	0.031 (4)	0.006 (3)	0.009 (3)	0.000 (3)
C28"	0.019 (3)	0.016 (3)	0.031 (4)	0.006 (3)	0.006 (3)	0.000 (3)
C28'	0.018 (3)	0.016 (3)	0.031 (4)	0.008 (2)	0.006 (3)	0.000 (3)
C29"	0.019 (3)	0.015 (3)	0.032 (4)	0.004 (3)	0.003 (3)	0.001 (3)
C29'	0.019 (2)	0.014 (3)	0.030 (4)	0.005 (2)	0.003 (3)	0.000 (3)
C30"	0.023 (3)	0.014 (3)	0.030 (4)	0.000 (3)	−0.003 (3)	−0.002 (3)
C30'	0.026 (3)	0.015 (3)	0.032 (4)	−0.001 (2)	−0.006 (3)	0.001 (3)
C31"	0.023 (3)	0.015 (3)	0.029 (4)	0.002 (3)	−0.006 (3)	−0.002 (3)
C31'	0.022 (3)	0.015 (3)	0.029 (4)	0.001 (2)	−0.005 (3)	−0.002 (3)
C32"	0.024 (3)	0.017 (4)	0.027 (3)	0.007 (3)	−0.004 (3)	−0.003 (3)
C32'	0.024 (3)	0.015 (4)	0.026 (3)	0.007 (3)	−0.005 (2)	−0.005 (3)
C33"	0.029 (3)	0.016 (4)	0.027 (3)	0.010 (3)	0.001 (3)	0.000 (3)
C33'	0.031 (3)	0.024 (4)	0.030 (3)	0.014 (3)	0.000 (3)	−0.004 (3)
C34"	0.027 (3)	0.019 (4)	0.030 (4)	0.009 (3)	0.007 (3)	−0.002 (3)
C34'	0.028 (3)	0.018 (3)	0.029 (3)	0.007 (3)	0.009 (3)	0.001 (3)
F1	0.0285 (19)	0.048 (2)	0.073 (3)	−0.0005 (16)	−0.0142 (19)	0.012 (2)
F2	0.039 (2)	0.036 (2)	0.078 (3)	−0.0018 (18)	−0.003 (2)	−0.002 (2)
F3	0.039 (2)	0.059 (2)	0.0292 (19)	−0.0038 (17)	0.0004 (16)	0.0076 (17)
F4	0.044 (2)	0.067 (3)	0.042 (2)	−0.005 (2)	0.0125 (18)	−0.003 (2)
B1	0.020 (3)	0.040 (4)	0.032 (4)	−0.006 (3)	0.000 (3)	0.002 (3)
F1'	0.047 (2)	0.028 (2)	0.117 (4)	0.0085 (18)	−0.038 (3)	−0.007 (3)
F2'	0.0337 (19)	0.035 (2)	0.053 (2)	−0.0053 (16)	−0.0059 (17)	−0.0016 (18)
F3'	0.055 (2)	0.099 (3)	0.027 (2)	−0.010 (2)	0.0137 (18)	−0.002 (2)
F4'	0.035 (2)	0.064 (3)	0.042 (2)	−0.0096 (18)	0.0121 (17)	0.011 (2)
B1'	0.023 (3)	0.037 (4)	0.025 (3)	−0.003 (3)	−0.001 (3)	0.004 (3)

### (4-Butyl-1-ethyl-1,2,4-triazol-5-ylidene)[(1,2,5,6-η)-cycloocta-1,5-diene](triphenylphosphane)iridium(I) tetrafluoridoborate Geometric parameters (Å, º)

**Table:**

Ir1—P1	2.3145 (13)	N2'—C2'	1.299 (8)
Ir1—C1	2.035 (5)	N3'—C1'	1.370 (7)
Ir1—C27	2.192 (6)	N3'—C2'	1.372 (7)
Ir1—C28	2.202 (6)	N3'—C5'	1.473 (7)
Ir1—C31	2.210 (5)	C2'—H2'	0.9500
Ir1—C32	2.209 (6)	C3'—H3'A	0.9900
P1—C9	1.846 (5)	C3'—H3'B	0.9900
P1—C15	1.817 (5)	C3'—C4'	1.529 (8)
P1—C21	1.830 (5)	C4'—H4'A	0.9800
N1—N2	1.381 (6)	C4'—H4'B	0.9800
N1—C1	1.352 (7)	C4'—H4'C	0.9800
N1—C3	1.460 (7)	C5'—H5'A	0.9900
N2—C2	1.304 (7)	C5'—H5'B	0.9900
N3—C1	1.364 (6)	C5'—C6'	1.531 (8)
N3—C2	1.365 (7)	C6'—H6'A	0.9900
N3—C5	1.470 (7)	C6'—H6'B	0.9900
C2—H2	0.9500	C6'—C7'	1.532 (9)
C3—H3A	0.9900	C7'—H7'A	0.9900
C3—H3B	0.9900	C7'—H7'B	0.9900
C3—C4	1.525 (8)	C7'—C8'	1.523 (9)
C4—H4A	0.9800	C8'—H8'A	0.9800
C4—H4B	0.9800	C8'—H8'B	0.9800
C4—H4C	0.9800	C8'—H8'C	0.9800
C5—H5A	0.9900	C9'—C10'	1.408 (7)
C5—H5B	0.9900	C9'—C14'	1.395 (7)
C5—C6	1.539 (7)	C10'—H10'	0.9500
C6—H6A	0.9900	C10'—C11'	1.385 (8)
C6—H6B	0.9900	C11'—H11'	0.9500
C6—C7	1.527 (10)	C11'—C12'	1.399 (9)
C7—H7A	0.9900	C12'—H12'	0.9500
C7—H7B	0.9900	C12'—C13'	1.389 (9)
C7—C8	1.524 (9)	C13'—H13'	0.9500
C8—H8A	0.9800	C13'—C14'	1.394 (8)
C8—H8B	0.9800	C14'—H14'	0.9500
C8—H8C	0.9800	C15'—C16'	1.406 (7)
C9—C10	1.384 (8)	C15'—C20'	1.387 (8)
C9—C14	1.403 (7)	C16'—H16'	0.9500
C10—H10	0.9500	C16'—C17'	1.384 (8)
C10—C11	1.387 (8)	C17'—H17'	0.9500
C11—H11	0.9500	C17'—C18'	1.389 (9)
C11—C12	1.370 (9)	C18'—H18'	0.9500
C12—H12	0.9500	C18'—C19'	1.376 (9)
C12—C13	1.388 (9)	C19'—H19'	0.9500
C13—H13	0.9500	C19'—C20'	1.393 (8)
C13—C14	1.393 (8)	C20'—H20'	0.9500
C14—H14	0.9500	C21'—C22'	1.393 (7)
C15—C16	1.386 (7)	C21'—C26'	1.398 (7)
C15—C20	1.424 (7)	C22'—H22'	0.9500
C16—H16	0.9500	C22'—C23'	1.394 (7)
C16—C17	1.395 (8)	C23'—H23'	0.9500
C17—H17	0.9500	C23'—C24'	1.393 (8)
C17—C18	1.392 (8)	C24'—H24'	0.9500
C18—H18	0.9500	C24'—C25'	1.387 (8)
C18—C19	1.392 (9)	C25'—H25'	0.9500
C19—H19	0.9500	C25'—C26'	1.393 (7)
C19—C20	1.385 (8)	C26'—H26'	0.9500
C20—H20	0.9500	C27"—H27"	1.0000
C21—C22	1.402 (7)	C27"—C28"	1.37 (4)
C21—C26	1.404 (8)	C27"—C34"	1.53 (3)
C22—H22	0.9500	C27'—H27'	1.0000
C22—C23	1.393 (8)	C27'—C28'	1.401 (18)
C23—H23	0.9500	C27'—C34'	1.515 (12)
C23—C24	1.382 (9)	C28"—H28"	1.0000
C24—H24	0.9500	C28"—C29"	1.48 (3)
C24—C25	1.390 (9)	C28'—H28'	1.0000
C25—H25	0.9500	C28'—C29'	1.536 (12)
C25—C26	1.388 (8)	C29"—H29A	0.9900
C26—H26	0.9500	C29"—H29B	0.9900
C27—H27	1.0000	C29"—C30"	1.47 (2)
C27—C28	1.386 (10)	C29'—H29C	0.9900
C27—C34	1.514 (8)	C29'—H29D	0.9900
C28—H28	1.0000	C29'—C30'	1.484 (12)
C28—C29	1.511 (9)	C30"—H30A	0.9900
C29—H29E	0.9900	C30"—H30B	0.9900
C29—H29F	0.9900	C30"—C31"	1.49 (3)
C29—C30	1.537 (8)	C30'—H30C	0.9900
C30—H30E	0.9900	C30'—H30D	0.9900
C30—H30F	0.9900	C30'—C31'	1.529 (13)
C30—C31	1.530 (8)	C31"—H31"	1.0000
C31—H31	1.0000	C31"—C32"	1.44 (4)
C31—C32	1.390 (9)	C31'—H31'	1.0000
C32—H32	1.0000	C31'—C32'	1.36 (2)
C32—C33	1.502 (8)	C32"—H32"	1.0000
C33—H33E	0.9900	C32"—C33"	1.53 (3)
C33—H33F	0.9900	C32'—H32'	1.0000
C33—C34	1.533 (8)	C32'—C33'	1.519 (13)
C34—H34E	0.9900	C33"—H33A	0.9900
C34—H34F	0.9900	C33"—H33B	0.9900
Ir1'—P1'	2.3154 (13)	C33"—C34"	1.45 (2)
Ir1'—C1'	2.034 (5)	C33'—H33C	0.9900
Ir1'—C27"	2.27 (3)	C33'—H33D	0.9900
Ir1'—C27'	2.160 (17)	C33'—C34'	1.470 (11)
Ir1'—C28"	2.16 (4)	C34"—H34A	0.9900
Ir1'—C28'	2.20 (2)	C34"—H34B	0.9900
Ir1'—C31"	2.18 (5)	C34'—H34C	0.9900
Ir1'—C31'	2.22 (3)	C34'—H34D	0.9900
Ir1'—C32"	2.24 (4)	F1—B1	1.384 (7)
Ir1'—C32'	2.18 (3)	F2—B1	1.382 (8)
P1'—C9'	1.822 (5)	F3—B1	1.377 (8)
P1'—C15'	1.842 (5)	F4—B1	1.412 (8)
P1'—C21'	1.829 (5)	F1'—B1'	1.362 (8)
N1'—N2'	1.375 (6)	F2'—B1'	1.380 (8)
N1'—C1'	1.354 (7)	F3'—B1'	1.381 (7)
N1'—C3'	1.453 (7)	F4'—B1'	1.394 (7)
			
C1—Ir1—P1	92.97 (14)	C1'—N1'—C3'	127.2 (4)
C1—Ir1—C27	93.7 (2)	C2'—N2'—N1'	103.5 (4)
C1—Ir1—C28	86.9 (2)	C1'—N3'—C2'	108.5 (5)
C1—Ir1—C31	157.2 (2)	C1'—N3'—C5'	126.7 (5)
C1—Ir1—C32	165.4 (2)	C2'—N3'—C5'	124.7 (5)
C27—Ir1—P1	157.38 (19)	N1'—C1'—Ir1'	124.2 (4)
C27—Ir1—C28	36.8 (2)	N1'—C1'—N3'	102.6 (4)
C27—Ir1—C31	86.8 (2)	N3'—C1'—Ir1'	133.2 (4)
C27—Ir1—C32	80.4 (2)	N2'—C2'—N3'	111.5 (5)
C28—Ir1—P1	165.48 (17)	N2'—C2'—H2'	124.2
C28—Ir1—C31	79.8 (2)	N3'—C2'—H2'	124.2
C28—Ir1—C32	96.0 (2)	N1'—C3'—H3'A	109.5
C31—Ir1—P1	95.25 (15)	N1'—C3'—H3'B	109.5
C32—Ir1—P1	87.78 (16)	N1'—C3'—C4'	110.9 (4)
C32—Ir1—C31	36.7 (2)	H3'A—C3'—H3'B	108.0
C9—P1—Ir1	119.21 (17)	C4'—C3'—H3'A	109.5
C15—P1—Ir1	113.04 (16)	C4'—C3'—H3'B	109.5
C15—P1—C9	100.9 (2)	C3'—C4'—H4'A	109.5
C15—P1—C21	105.0 (2)	C3'—C4'—H4'B	109.5
C21—P1—Ir1	111.86 (17)	C3'—C4'—H4'C	109.5
C21—P1—C9	105.5 (2)	H4'A—C4'—H4'B	109.5
N2—N1—C3	119.0 (4)	H4'A—C4'—H4'C	109.5
C1—N1—N2	113.9 (4)	H4'B—C4'—H4'C	109.5
C1—N1—C3	127.1 (4)	N3'—C5'—H5'A	109.5
C2—N2—N1	102.7 (4)	N3'—C5'—H5'B	109.5
C1—N3—C2	108.5 (4)	N3'—C5'—C6'	110.8 (5)
C1—N3—C5	127.5 (4)	H5'A—C5'—H5'B	108.1
C2—N3—C5	123.9 (5)	C6'—C5'—H5'A	109.5
N1—C1—Ir1	124.1 (4)	C6'—C5'—H5'B	109.5
N1—C1—N3	102.8 (4)	C5'—C6'—H6'A	109.2
N3—C1—Ir1	133.0 (4)	C5'—C6'—H6'B	109.2
N2—C2—N3	112.1 (5)	C5'—C6'—C7'	112.2 (5)
N2—C2—H2	123.9	H6'A—C6'—H6'B	107.9
N3—C2—H2	123.9	C7'—C6'—H6'A	109.2
N1—C3—H3A	109.3	C7'—C6'—H6'B	109.2
N1—C3—H3B	109.3	C6'—C7'—H7'A	109.4
N1—C3—C4	111.5 (5)	C6'—C7'—H7'B	109.4
H3A—C3—H3B	108.0	H7'A—C7'—H7'B	108.0
C4—C3—H3A	109.3	C8'—C7'—C6'	111.2 (6)
C4—C3—H3B	109.3	C8'—C7'—H7'A	109.4
C3—C4—H4A	109.5	C8'—C7'—H7'B	109.4
C3—C4—H4B	109.5	C7'—C8'—H8'A	109.5
C3—C4—H4C	109.5	C7'—C8'—H8'B	109.5
H4A—C4—H4B	109.5	C7'—C8'—H8'C	109.5
H4A—C4—H4C	109.5	H8'A—C8'—H8'B	109.5
H4B—C4—H4C	109.5	H8'A—C8'—H8'C	109.5
N3—C5—H5A	109.4	H8'B—C8'—H8'C	109.5
N3—C5—H5B	109.4	C10'—C9'—P1'	118.6 (4)
N3—C5—C6	111.0 (4)	C14'—C9'—P1'	121.7 (4)
H5A—C5—H5B	108.0	C14'—C9'—C10'	119.7 (5)
C6—C5—H5A	109.4	C9'—C10'—H10'	120.0
C6—C5—H5B	109.4	C11'—C10'—C9'	120.0 (5)
C5—C6—H6A	109.4	C11'—C10'—H10'	120.0
C5—C6—H6B	109.4	C10'—C11'—H11'	119.9
H6A—C6—H6B	108.0	C10'—C11'—C12'	120.2 (6)
C7—C6—C5	111.2 (5)	C12'—C11'—H11'	119.9
C7—C6—H6A	109.4	C11'—C12'—H12'	120.2
C7—C6—H6B	109.4	C13'—C12'—C11'	119.6 (6)
C6—C7—H7A	109.3	C13'—C12'—H12'	120.2
C6—C7—H7B	109.3	C12'—C13'—H13'	119.7
H7A—C7—H7B	108.0	C12'—C13'—C14'	120.7 (6)
C8—C7—C6	111.6 (6)	C14'—C13'—H13'	119.7
C8—C7—H7A	109.3	C9'—C14'—H14'	120.1
C8—C7—H7B	109.3	C13'—C14'—C9'	119.7 (5)
C7—C8—H8A	109.5	C13'—C14'—H14'	120.1
C7—C8—H8B	109.5	C16'—C15'—P1'	116.3 (4)
C7—C8—H8C	109.5	C20'—C15'—P1'	124.7 (4)
H8A—C8—H8B	109.5	C20'—C15'—C16'	118.9 (5)
H8A—C8—H8C	109.5	C15'—C16'—H16'	119.8
H8B—C8—H8C	109.5	C17'—C16'—C15'	120.4 (5)
C10—C9—P1	124.7 (4)	C17'—C16'—H16'	119.8
C10—C9—C14	119.0 (5)	C16'—C17'—H17'	120.0
C14—C9—P1	116.2 (4)	C16'—C17'—C18'	120.0 (6)
C9—C10—H10	120.1	C18'—C17'—H17'	120.0
C9—C10—C11	119.8 (5)	C17'—C18'—H18'	120.0
C11—C10—H10	120.1	C19'—C18'—C17'	120.0 (6)
C10—C11—H11	119.4	C19'—C18'—H18'	120.0
C12—C11—C10	121.3 (6)	C18'—C19'—H19'	119.7
C12—C11—H11	119.4	C18'—C19'—C20'	120.5 (6)
C11—C12—H12	120.0	C20'—C19'—H19'	119.7
C11—C12—C13	120.0 (6)	C15'—C20'—C19'	120.2 (6)
C13—C12—H12	120.0	C15'—C20'—H20'	119.9
C12—C13—H13	120.3	C19'—C20'—H20'	119.9
C12—C13—C14	119.3 (6)	C22'—C21'—P1'	118.7 (4)
C14—C13—H13	120.3	C22'—C21'—C26'	118.6 (5)
C9—C14—H14	119.7	C26'—C21'—P1'	122.2 (4)
C13—C14—C9	120.6 (5)	C21'—C22'—H22'	119.5
C13—C14—H14	119.7	C21'—C22'—C23'	120.9 (5)
C16—C15—P1	122.3 (4)	C23'—C22'—H22'	119.5
C16—C15—C20	119.2 (5)	C22'—C23'—H23'	120.0
C20—C15—P1	118.5 (4)	C24'—C23'—C22'	119.9 (5)
C15—C16—H16	119.9	C24'—C23'—H23'	120.0
C15—C16—C17	120.2 (5)	C23'—C24'—H24'	120.2
C17—C16—H16	119.9	C25'—C24'—C23'	119.5 (5)
C16—C17—H17	119.8	C25'—C24'—H24'	120.2
C18—C17—C16	120.5 (5)	C24'—C25'—H25'	119.8
C18—C17—H17	119.8	C24'—C25'—C26'	120.5 (5)
C17—C18—H18	120.1	C26'—C25'—H25'	119.8
C17—C18—C19	119.9 (5)	C21'—C26'—H26'	119.8
C19—C18—H18	120.1	C25'—C26'—C21'	120.4 (5)
C18—C19—H19	119.9	C25'—C26'—H26'	119.8
C20—C19—C18	120.2 (5)	Ir1'—C27"—H27"	115.1
C20—C19—H19	119.9	C28"—C27"—Ir1'	68 (2)
C15—C20—H20	120.0	C28"—C27"—H27"	115.1
C19—C20—C15	120.1 (5)	C28"—C27"—C34"	125 (2)
C19—C20—H20	120.0	C34"—C27"—Ir1'	109.6 (15)
C22—C21—P1	121.5 (4)	C34"—C27"—H27"	115.1
C22—C21—C26	119.1 (5)	Ir1'—C27'—H27'	113.7
C26—C21—P1	118.5 (4)	C28'—C27'—Ir1'	72.8 (13)
C21—C22—H22	120.2	C28'—C27'—H27'	113.7
C23—C22—C21	119.5 (6)	C28'—C27'—C34'	125.8 (13)
C23—C22—H22	120.2	C34'—C27'—Ir1'	109.5 (9)
C22—C23—H23	119.4	C34'—C27'—H27'	113.7
C24—C23—C22	121.1 (6)	Ir1'—C28"—H28"	113.6
C24—C23—H23	119.4	C27"—C28"—Ir1'	76 (2)
C23—C24—H24	120.2	C27"—C28"—H28"	113.6
C23—C24—C25	119.5 (5)	C27"—C28"—C29"	124 (2)
C25—C24—H24	120.2	C29"—C28"—Ir1'	109 (2)
C24—C25—H25	119.9	C29"—C28"—H28"	113.6
C26—C25—C24	120.3 (6)	Ir1'—C28'—H28'	113.7
C26—C25—H25	119.9	C27'—C28'—Ir1'	69.7 (12)
C21—C26—H26	119.8	C27'—C28'—H28'	113.7
C25—C26—C21	120.4 (5)	C27'—C28'—C29'	125.5 (13)
C25—C26—H26	119.8	C29'—C28'—Ir1'	112.6 (12)
Ir1—C27—H27	113.2	C29'—C28'—H28'	113.7
C28—C27—Ir1	72.0 (3)	C28"—C29"—H29A	107.7
C28—C27—H27	113.2	C28"—C29"—H29B	107.7
C28—C27—C34	125.8 (6)	H29A—C29"—H29B	107.1
C34—C27—Ir1	112.5 (4)	C30"—C29"—C28"	118 (2)
C34—C27—H27	113.2	C30"—C29"—H29A	107.7
Ir1—C28—H28	113.9	C30"—C29"—H29B	107.7
C27—C28—Ir1	71.3 (4)	C28'—C29'—H29C	108.2
C27—C28—H28	113.9	C28'—C29'—H29D	108.2
C27—C28—C29	126.2 (6)	H29C—C29'—H29D	107.3
C29—C28—Ir1	109.4 (4)	C30'—C29'—C28'	116.4 (12)
C29—C28—H28	113.9	C30'—C29'—H29C	108.2
C28—C29—H29E	109.1	C30'—C29'—H29D	108.2
C28—C29—H29F	109.1	C29"—C30"—H30A	107.9
C28—C29—C30	112.5 (5)	C29"—C30"—H30B	107.9
H29E—C29—H29F	107.8	C29"—C30"—C31"	118 (2)
C30—C29—H29E	109.1	H30A—C30"—H30B	107.2
C30—C29—H29F	109.1	C31"—C30"—H30A	107.9
C29—C30—H30E	109.2	C31"—C30"—H30B	107.9
C29—C30—H30F	109.2	C29'—C30'—H30C	108.8
H30E—C30—H30F	107.9	C29'—C30'—H30D	108.8
C31—C30—C29	112.2 (4)	C29'—C30'—C31'	113.9 (14)
C31—C30—H30E	109.2	H30C—C30'—H30D	107.7
C31—C30—H30F	109.2	C31'—C30'—H30C	108.8
Ir1—C31—H31	113.7	C31'—C30'—H30D	108.8
C30—C31—Ir1	112.7 (4)	Ir1'—C31"—H31"	115.5
C30—C31—H31	113.7	C30"—C31"—Ir1'	108 (2)
C32—C31—Ir1	71.6 (3)	C30"—C31"—H31"	115.5
C32—C31—C30	124.2 (5)	C32"—C31"—Ir1'	73 (3)
C32—C31—H31	113.7	C32"—C31"—C30"	122 (3)
Ir1—C32—H32	114.4	C32"—C31"—H31"	115.5
C31—C32—Ir1	71.7 (3)	Ir1'—C31'—H31'	113.0
C31—C32—H32	114.4	C30'—C31'—Ir1'	111.3 (15)
C31—C32—C33	125.0 (6)	C30'—C31'—H31'	113.0
C33—C32—Ir1	108.8 (4)	C32'—C31'—Ir1'	70.5 (17)
C33—C32—H32	114.4	C32'—C31'—C30'	127.8 (18)
C32—C33—H33E	108.8	C32'—C31'—H31'	113.0
C32—C33—H33F	108.8	Ir1'—C32"—H32"	114.2
C32—C33—C34	113.7 (5)	C31"—C32"—Ir1'	69 (3)
H33E—C33—H33F	107.7	C31"—C32"—H32"	114.2
C34—C33—H33E	108.8	C31"—C32"—C33"	124 (3)
C34—C33—H33F	108.8	C33"—C32"—Ir1'	113 (2)
C27—C34—C33	113.7 (5)	C33"—C32"—H32"	114.2
C27—C34—H34E	108.8	Ir1'—C32'—H32'	114.0
C27—C34—H34F	108.8	C31'—C32'—Ir1'	73.7 (17)
C33—C34—H34E	108.8	C31'—C32'—H32'	114.0
C33—C34—H34F	108.8	C31'—C32'—C33'	122.4 (15)
H34E—C34—H34F	107.7	C33'—C32'—Ir1'	112.3 (13)
C1'—Ir1'—P1'	91.62 (15)	C33'—C32'—H32'	114.0
C1'—Ir1'—C27"	89.5 (8)	C32"—C33"—H33A	108.3
C1'—Ir1'—C27'	88.2 (5)	C32"—C33"—H33B	108.3
C1'—Ir1'—C28"	91.6 (8)	H33A—C33"—H33B	107.4
C1'—Ir1'—C28'	95.3 (5)	C34"—C33"—C32"	116 (2)
C1'—Ir1'—C31"	164.0 (9)	C34"—C33"—H33A	108.3
C1'—Ir1'—C31'	168.4 (5)	C34"—C33"—H33B	108.3
C1'—Ir1'—C32"	157.7 (9)	C32'—C33'—H33C	108.7
C1'—Ir1'—C32'	155.3 (5)	C32'—C33'—H33D	108.7
C27"—Ir1'—P1'	172.7 (6)	H33C—C33'—H33D	107.6
C27'—Ir1'—P1'	166.2 (3)	C34'—C33'—C32'	114.0 (12)
C27'—Ir1'—C28'	37.5 (5)	C34'—C33'—H33C	108.7
C27'—Ir1'—C31'	93.6 (7)	C34'—C33'—H33D	108.7
C27'—Ir1'—C32'	80.6 (6)	C27"—C34"—H34A	107.6
C28"—Ir1'—P1'	151.1 (8)	C27"—C34"—H34B	107.6
C28"—Ir1'—C27"	36.0 (9)	C33"—C34"—C27"	118.8 (17)
C28"—Ir1'—C31"	82.6 (13)	C33"—C34"—H34A	107.6
C28"—Ir1'—C32"	90.3 (11)	C33"—C34"—H34B	107.6
C28'—Ir1'—P1'	156.0 (4)	H34A—C34"—H34B	107.0
C28'—Ir1'—C31'	79.5 (7)	C27'—C34'—H34C	108.4
C31"—Ir1'—P1'	86.5 (11)	C27'—C34'—H34D	108.4
C31"—Ir1'—C27"	94.3 (12)	C33'—C34'—C27'	115.4 (10)
C31"—Ir1'—C32"	37.8 (11)	C33'—C34'—H34C	108.4
C31'—Ir1'—P1'	89.3 (6)	C33'—C34'—H34D	108.4
C32"—Ir1'—P1'	97.4 (8)	H34C—C34'—H34D	107.5
C32"—Ir1'—C27"	79.1 (11)	F1—B1—F4	109.2 (6)
C32'—Ir1'—P1'	94.2 (5)	F2—B1—F1	110.9 (6)
C32'—Ir1'—C28'	89.1 (6)	F2—B1—F4	109.2 (5)
C32'—Ir1'—C31'	35.9 (5)	F3—B1—F1	109.5 (5)
C9'—P1'—Ir1'	112.40 (16)	F3—B1—F2	111.1 (6)
C9'—P1'—C15'	101.7 (2)	F3—B1—F4	106.9 (6)
C9'—P1'—C21'	104.4 (2)	F1'—B1'—F2'	110.6 (5)
C15'—P1'—Ir1'	116.94 (17)	F1'—B1'—F3'	110.6 (6)
C21'—P1'—Ir1'	113.86 (17)	F1'—B1'—F4'	109.7 (6)
C21'—P1'—C15'	106.1 (2)	F2'—B1'—F3'	109.1 (5)
N2'—N1'—C3'	118.8 (4)	F2'—B1'—F4'	110.3 (6)
C1'—N1'—N2'	113.8 (4)	F3'—B1'—F4'	106.5 (5)
			
Ir1—P1—C9—C10	121.4 (5)	Ir1'—C27'—C28'—C29'	−103.8 (19)
Ir1—P1—C9—C14	−61.6 (5)	Ir1'—C27'—C34'—C33'	32.5 (12)
Ir1—P1—C15—C16	−2.1 (5)	Ir1'—C28"—C29"—C30"	−7 (2)
Ir1—P1—C15—C20	177.8 (4)	Ir1'—C28'—C29'—C30'	1.4 (15)
Ir1—P1—C21—C22	101.8 (4)	Ir1'—C31"—C32"—C33"	−105 (4)
Ir1—P1—C21—C26	−67.4 (4)	Ir1'—C31'—C32'—C33'	−106 (2)
Ir1—C27—C28—C29	−100.8 (6)	Ir1'—C32"—C33"—C34"	2 (2)
Ir1—C27—C34—C33	−9.0 (7)	Ir1'—C32'—C33'—C34'	5.9 (13)
Ir1—C28—C29—C30	−40.2 (6)	P1'—C9'—C10'—C11'	−177.7 (4)
Ir1—C31—C32—C33	−100.5 (6)	P1'—C9'—C14'—C13'	178.5 (4)
Ir1—C32—C33—C34	−36.7 (6)	P1'—C15'—C16'—C17'	−176.3 (5)
P1—C9—C10—C11	177.9 (5)	P1'—C15'—C20'—C19'	175.6 (5)
P1—C9—C14—C13	−177.8 (5)	P1'—C21'—C22'—C23'	−175.7 (4)
P1—C15—C16—C17	−178.7 (4)	P1'—C21'—C26'—C25'	174.6 (4)
P1—C15—C20—C19	177.9 (4)	N1'—N2'—C2'—N3'	−0.5 (7)
P1—C21—C22—C23	−170.6 (4)	N2'—N1'—C1'—Ir1'	−179.2 (4)
P1—C21—C26—C25	171.6 (4)	N2'—N1'—C1'—N3'	0.9 (6)
N1—N2—C2—N3	0.2 (6)	N2'—N1'—C3'—C4'	−64.2 (7)
N2—N1—C1—Ir1	−178.0 (3)	N3'—C5'—C6'—C7'	−173.8 (5)
N2—N1—C1—N3	−1.6 (6)	C1'—N1'—N2'—C2'	−0.3 (6)
N2—N1—C3—C4	66.1 (6)	C1'—N1'—C3'—C4'	111.1 (6)
N3—C5—C6—C7	168.7 (5)	C1'—N3'—C2'—N2'	1.1 (7)
C1—N1—N2—C2	0.9 (6)	C1'—N3'—C5'—C6'	−113.5 (6)
C1—N1—C3—C4	−113.0 (6)	C2'—N3'—C1'—Ir1'	179.0 (4)
C1—N3—C2—N2	−1.2 (7)	C2'—N3'—C1'—N1'	−1.2 (6)
C1—N3—C5—C6	117.5 (6)	C2'—N3'—C5'—C6'	65.0 (7)
C2—N3—C1—Ir1	177.6 (4)	C3'—N1'—N2'—C2'	175.6 (5)
C2—N3—C1—N1	1.6 (6)	C3'—N1'—C1'—Ir1'	5.3 (7)
C2—N3—C5—C6	−65.0 (7)	C3'—N1'—C1'—N3'	−174.6 (5)
C3—N1—N2—C2	−178.3 (5)	C5'—N3'—C1'—Ir1'	−2.3 (8)
C3—N1—C1—Ir1	1.1 (7)	C5'—N3'—C1'—N1'	177.5 (5)
C3—N1—C1—N3	177.5 (5)	C5'—N3'—C2'—N2'	−177.6 (5)
C5—N3—C1—Ir1	−4.7 (8)	C5'—C6'—C7'—C8'	178.7 (6)
C5—N3—C1—N1	179.4 (5)	C9'—P1'—C15'—C16'	−50.2 (5)
C5—N3—C2—N2	−179.0 (5)	C9'—P1'—C15'—C20'	133.5 (5)
C5—C6—C7—C8	−177.1 (5)	C9'—P1'—C21'—C22'	−173.0 (4)
C9—P1—C15—C16	−130.6 (5)	C9'—P1'—C21'—C26'	15.2 (5)
C9—P1—C15—C20	49.4 (5)	C9'—C10'—C11'—C12'	−1.2 (9)
C9—P1—C21—C22	−127.2 (4)	C10'—C9'—C14'—C13'	−0.5 (8)
C9—P1—C21—C26	63.6 (5)	C10'—C11'—C12'—C13'	0.2 (10)
C9—C10—C11—C12	−1.0 (9)	C11'—C12'—C13'—C14'	0.7 (10)
C10—C9—C14—C13	−0.6 (8)	C12'—C13'—C14'—C9'	−0.5 (9)
C10—C11—C12—C13	0.8 (10)	C14'—C9'—C10'—C11'	1.3 (8)
C11—C12—C13—C14	−0.5 (10)	C15'—P1'—C9'—C10'	−47.2 (5)
C12—C13—C14—C9	0.4 (9)	C15'—P1'—C9'—C14'	133.8 (5)
C14—C9—C10—C11	0.9 (8)	C15'—P1'—C21'—C22'	−66.0 (5)
C15—P1—C9—C10	−114.3 (5)	C15'—P1'—C21'—C26'	122.2 (4)
C15—P1—C9—C14	62.8 (4)	C15'—C16'—C17'—C18'	0.4 (9)
C15—P1—C21—C22	−21.2 (5)	C16'—C15'—C20'—C19'	−0.6 (9)
C15—P1—C21—C26	169.6 (4)	C16'—C17'—C18'—C19'	−0.6 (10)
C15—C16—C17—C18	0.1 (9)	C17'—C18'—C19'—C20'	0.2 (10)
C16—C15—C20—C19	−2.1 (8)	C18'—C19'—C20'—C15'	0.4 (10)
C16—C17—C18—C19	−0.8 (9)	C20'—C15'—C16'—C17'	0.2 (8)
C17—C18—C19—C20	0.0 (9)	C21'—P1'—C9'—C10'	63.0 (5)
C18—C19—C20—C15	1.4 (9)	C21'—P1'—C9'—C14'	−116.0 (5)
C20—C15—C16—C17	1.3 (8)	C21'—P1'—C15'—C16'	−159.1 (4)
C21—P1—C9—C10	−5.3 (6)	C21'—P1'—C15'—C20'	24.6 (6)
C21—P1—C9—C14	171.8 (4)	C21'—C22'—C23'—C24'	2.2 (8)
C21—P1—C15—C16	120.0 (4)	C22'—C21'—C26'—C25'	2.7 (8)
C21—P1—C15—C20	−60.0 (4)	C22'—C23'—C24'—C25'	0.3 (8)
C21—C22—C23—C24	0.2 (8)	C23'—C24'—C25'—C26'	−1.1 (8)
C22—C21—C26—C25	2.2 (8)	C24'—C25'—C26'—C21'	−0.4 (8)
C22—C23—C24—C25	0.5 (9)	C26'—C21'—C22'—C23'	−3.6 (8)
C23—C24—C25—C26	0.2 (9)	C27"—C28"—C29"—C30"	79 (4)
C24—C25—C26—C21	−1.6 (8)	C27'—C28'—C29'—C30'	82 (2)
C26—C21—C22—C23	−1.5 (7)	C28"—C27"—C34"—C33"	−55 (3)
C27—C28—C29—C30	40.3 (8)	C28"—C29"—C30"—C31"	−9 (3)
C28—C27—C34—C33	−92.5 (7)	C28'—C27'—C34'—C33'	−50 (2)
C28—C29—C30—C31	36.3 (7)	C28'—C29'—C30'—C31'	−19.6 (16)
C29—C30—C31—Ir1	−14.4 (6)	C29"—C30"—C31"—Ir1'	20 (2)
C29—C30—C31—C32	−97.0 (7)	C29"—C30"—C31"—C32"	−60 (5)
C30—C31—C32—Ir1	105.5 (5)	C29'—C30'—C31'—Ir1'	28.0 (15)
C30—C31—C32—C33	5.0 (9)	C29'—C30'—C31'—C32'	−53 (3)
C31—C32—C33—C34	43.7 (8)	C30"—C31"—C32"—Ir1'	101 (4)
C32—C33—C34—C27	31.0 (7)	C30"—C31"—C32"—C33"	−4 (6)
C34—C27—C28—Ir1	105.1 (6)	C30'—C31'—C32'—Ir1'	102 (3)
C34—C27—C28—C29	4.4 (10)	C30'—C31'—C32'—C33'	−4 (4)
Ir1'—P1'—C9'—C10'	−173.1 (4)	C31"—C32"—C33"—C34"	82 (4)
Ir1'—P1'—C9'—C14'	7.9 (5)	C31'—C32'—C33'—C34'	90 (3)
Ir1'—P1'—C15'—C16'	72.6 (4)	C32"—C33"—C34"—C27"	−16 (2)
Ir1'—P1'—C15'—C20'	−103.7 (5)	C32'—C33'—C34'—C27'	−25.7 (14)
Ir1'—P1'—C21'—C22'	64.1 (4)	C34"—C27"—C28"—Ir1'	99 (2)
Ir1'—P1'—C21'—C26'	−107.8 (4)	C34"—C27"—C28"—C29"	−5 (5)
Ir1'—C27"—C28"—C29"	−104 (3)	C34'—C27'—C28'—Ir1'	102.0 (15)
Ir1'—C27"—C34"—C33"	21 (2)	C34'—C27'—C28'—C29'	−2 (3)

### (4-Butyl-1-ethyl-1,2,4-triazol-5-ylidene)[(1,2,5,6-η)-cycloocta-1,5-diene](triphenylphosphane)iridium(I) tetrafluoridoborate Hydrogen-bond geometry (Å, º)

**Table:**

*D*—H···*A*	*D*—H	H···*A*	*D*···*A*	*D*—H···*A*
C2—H2···F4′	0.95	2.35	3.255 (7)	158
C4—H4*B*···F2^i^	0.98	2.49	3.352 (8)	147
C2′—H2′···F1^ii^	0.95	2.21	3.014 (6)	141
C11′—H11′···F4^iii^	0.95	2.46	3.363 (7)	160
C30′—H30*D*···F3′^iii^	0.99	2.49	3.355 (15)	146

Symmetry codes: (i) *x*, −*y*+1, *z*+1/2; (ii) *x*−1, −*y*+1, *z*−1/2; (iii) *x*, −*y*+1, *z*−1/2.
